# On the Nature and Treatment of Stomach and Renal Diseases; Being an Inquiry into the Connexion of Diabetes, Calculus, and Other Affections of the Kidney and Bladder, with Indigestion

**Published:** 1844-03

**Authors:** 


ART. V.—*On the Nature and Treatment of Stomach and Renal Diseases;* being an inquiry into the *connexion of Diabetes, Calculus, and other Affections of the Kidney and Bladder, with Indigestion.* By WILLIAM PROUT, M.D., F.R.S., Fellow of the Royal College of Physicians. *From the 4th revised London Edition, with plates.* Philadelphia: Lea & Blanchard. 1843. 8mo. pp. 465.

This treatise has been now many years before the public, and no more satisfactory proof of its excellence could be given, than the fact that it has held a place, so long, among the standard works of the profession. It would seem quite an unnecessary service to commend to our readers a work, the reputation of which is so well established, and altogether out of time to undertake an analysis of its contents. Doubtless this would be true, if Dr. Prout's valuable labors were as well known in this region of our country as the present re-print of his book promises soon to make them. But at present we doubt, whether this volume is to be found generally in the libraries of our physicians. At all events, we have not often met with it in private libraries, and since it treats with singular clearness and ability of a class of affections, unsurpassed in importance by any other, and which often perplex the practitioner by the obscurity of their diagnosis, we have concluded that we could not appropriate a few pages more profitably, even at this late day, than to an analytical review of this work.

Dr. Prout divides the proximate alimentary principles into four classess, viz: the Aqueous, the Saccharine, the Albuminous, and the Oleaginous, and in conformity with this constistitution, he considers the pathology of the assimilating processes under four similar heads. The first part of his treatise comprises the following subjects:

CHAP. I. General observations on the Pathology of Aqueous assimilation and secretion.

*Section a.* Of the relation of Fluids to the assimilating processes in Health and in Disease.

*b.* Of the relation of Fluids to Nephritic operations in Health and in Disease.

Chap. II. General observations on the Pathology of Saccharine assimilation and secretion.

*Section a.* Of Saccharine urine. *Diabetes.*

*b.* Of Oxalic acid; Oxalate of Lime.

*c.* Of Lactic acid.

Chap. III. General observations on the Pathology of Albuminous assimilation and secretion.

*Section a.* Of an Excess and Deficiency of Urea.

*b.* Of Albuminous urine.

*c.* Of Lithic acid.

*d.* Of Cystic Oxide.

Chap. IV. General observations on the Pathology of Oleaginous assimilation and secretion.

*Section a.* Of an Excess and Deficiency of fat.

*b.* Of Cholesterine and its Deposites, &c.

The important class of diseases, arising from the Mineral matters incidental to the proximate animal principles, will be separately considered under the head of

Chap. V. General observations on the Pathology of the Incidental Mineral matters entering into the composition of organized bodies.

It is only lately that the important truth has come to be generally recognized, that the bodies of animals consist essentially of the proximate alimentary principles by which their existence is maintained. But these matters are greatly modified by the assimilating organs, and hence it follows, that any derangement of their function must show itself in an unhealthy state of the secretions, especially of the liver and kidneys. Thus, in diabetes, sugar is found not only in the urine, but also in the blood, which in healthy individuals never contains it, the stomach having lost the faculty of assimilating this principle. The first step in the derangements producing this disease, consists, therefore, in the greater or less destruction of this faculty. The saccharine state of the urine is regarded by Dr. Prout as the characteristic symptom of this affection, to the consideration of which the first section of his second chapter is devoted.

Diabetic urine is transparent, of a pale straw or greenish color, of a specific gravity greater than water, being sometimes as high as 1.055, and containg in a wine pint very nearly two ounces of solid extract. The quantity of urine voided is sometimes enormous, thirty pints having been discharged in twenty-four hours, for weeks and even months together, in some cases. The tongue becomes loaded with a white frothy mucus, the mouth clammy, while the appetite remains unimpaired, or is even better than ordinary; but under the wasting discharge of urine, the patient daily loses flesh and strength, the skin grows dry, a sense of chilliness and fatigue is felt, accompanied by dull pain in the back and loins and in the lower extremities. If the disease be recognized and properly treated, at this stage, its progress may in many cases be arrested, and health may be restored. Unfortunately, however, it is apt to be overlooked, or mistaken, and under an improper treatment advances to an incurable stage. The urine is voided in still greater abundance, the thirst increases, the mouth is dry and parched, the tongue becomes preternaturally clean and red, an uneasiness is felt in the region of the stomach, attended with a sense of sinking, and incessant craving after food and drinks. The mind suffers, and grows fretful and suspicious, the pulse becomes feeble and accelerated, some important organ is involved in disease, the breathing becomes disturbed, a cough comes on, and phthisis is finally developed, or else the patient grows comatose and sinks under symptoms of cerebral disease. Such is the progress of diabetes in its simplest form.

Diabetes seems to be peculiar to mankind. In horses and other animals the urine is often much increased in quantity, but is never saccharine. A predisposition to the disease is frequently inherited, and when this is present many exciting causes may bring it out, such as exposure to cold, attacks of gout and rheumatism, the drinking of cold fluids while heated, mental distress, concussions or injuries of the back, and cutaneous diseases. It almost "always accompanies carbuncles, and malignant boils allied to carbuncles."

The prognosis is generally unfavorable, except, as mentioned in the early stage of the disease. Much of the treatment consists in regulating the diet. Saccharine matters, and those convertible into sugar are to be avoided as much as possible. It is to be carefully guarded in quantity, as well as quality. The same remark applies equally to the use of fluids, and the management of drinks in this disease is often felt to be one of the points of the greatest difficulty. Water is to be sparingly used, and milk and animal decoctions to be taken instead. By regimen the quantity of urine is to be diminished, and its character improved. As to medication our author observes:

"We have already stated, that no specific remedy is known for the essential symptom of diabetes; in other words, we can no more directly restore the paralyzed function of the assimilating organs, than we can directly restore the paralyzed function of an arm or of a leg. The medical treatment of diabetes, therefore, must be conducted on general principles; and consequently must not only vary according to the degree and complication of the affection; but according to the general state of health and the peculiar constitution of the patient."

General blood-letting is demanded in recent and acute cases, and may be repeated according to circumstances; and in most cases local bleedings from the epigastric region are beneficial. Purgatives are much relied on by some, but in the estimation of our author, they are most useful when limited to the regulation of the bowels. The action of the skin is to be restored, if practicable, by the use of diaphoretics. Tonics, sedatives and astringents are also prescribed, with many other remedies not referrible to any of the preceding classes. Among the most recent and active of these may be mentioned creasote, prussic acid, colchicum, strychnine, and iodide of potassium, which are employed empirically. When complicated with other diseases, as, for example, with disorder of the liver, such complications must be taken into account, and the treatment modified accordingly.

In connexion with the hepatic complaint often associated with diabetes, Dr. Prout takes occasion to protest against the abuse of mercury, which he believes to be very great in the treatment of such affections. Mercury, he insists, "ought never to be administered for those slight deviations from health which can be readily removed by safer expedients," and should be cautiously administered to those on "whose constitution its effects have not yet been ascertained." He reprobates the practice of "a calomel pill at night, and a black dose in the morning, for all diseases and all kinds of constitutions—from the congested liver of the overgorged alderman, to the torpid liver of the weak and indolent female." And, we suppose, few of his readers will have the hardihood to differ with him. It must be admitted, that some discrimination is necessary in the practice of physic.

Dr. Prout closes his remarks on diabetes with an account of a diuresis which occurs in children soon after the period of weaning, and which may be characterized by albuminous matters in the urine, by an excess or deficiency of urea, or by saccharine matter. At first the urine is scanty and high colored, but as the disease proceeds the quantity rapidly increases; the bowels are disordered; the skin harsh and dry; and the pulse quick and irritable. If badly treated, it terminates in organic lesion of the kidneys. It is in all its forms a disease of a formidable character, and when it has assumed its worst aspect may be considered hopeless. The treatment in it consists in regulating the diet; in removing the sufferer to a pure bracing air, and carefully attending to the state of the bowels. Of diet, milk forms the most eligible article.

We pass, with a single quotation, over the section on the *oxalic acid diathesis*, which our author supposes to prevail in the class of diseases now under consideration:

"The presence of oxalic acid in the system, as far as the primary assimilating processes are concerned, arises from one of two causes, which, for want of a better name, we term *proximate* causes; viz: the non-assimilation of oxalic acid taken as food; and the mal-assimilation of saccharine aliments, and in extreme cases, perhaps, of albuminous and oleaginous aliments. The first of these causes may exist without the second, and may even give occasion to the formation of a nephritic oxalate of lime attack, without producing, in a marked degree at least, the constitutional symptoms usually present in this diathesis; of which I have seen instances. It is probable that the first cause operates principally in those in whom the *converting* function of the stomach is *deficient in power;* and in whom, at the same time, there is a predisposition to the oxalic acid diathesis; for there is every reason to believe that the perfectly healthy stomach can convert small quantities of the oxalic acid when mixed with the articles of food. The second cause consists in something more than mere weakness; there is in this case a *positive derangement of the converting function* of the stomach. These two conditions, however, of the converting functions of the stomach, may be supposed to be so nearly allied, as not only in some instances to co-exist, but to pass into each other."

The next section treats of *lactic acid* as one of the modifications of these derangements. This acid, with the muriatic, is always present in the stomach during the reducing process, derived partly from the blood, and partly from the imperfectly assimilated food. The predominance of the muriatic acid seems to be connected with an inflammatory state of the system—that of the lactic acid, with a state of irritation. Thus the muriatic is found in dyspeptics of a plethoric or gouty habit, and the lactic in those of a delicate or nervous constitution. Other acids accompany these two, when they abound, as the oxalic, carbonic, butyric, &c., but that product which proves the most troublesome is nitrogen, which, in nervous subjects, "is occasionally developed from the stomach in enormous quantities," and which being less stimulating than any other of the gaseous principles is apt to be retained, thus distending the stomach, and adding greatly to the miseries of the patient. The muriatic acid, derived from the muriate of soda existing in the blood, when it passes from the stomach into the duodenum, is probably neutralized by the soda of the bile, when that secretion is healthy. But the lactic acid must descend into the intestines uncombined, since there can be no equivalent of alkali in the duodenum to neutralize it, and is taken up by the lacteals, or passes into the inferior bowels, giving rise to a variety of unpleasant symptoms.

The natural condition of the intestinal canal, Dr. Prout conceives, "with the exception, perhaps, of the cœcum, is either neutral, or occasionally verges towards slight acidity on the one hand, and slight alkalescence on the other." Disturbance, therefore, must be the result of the passage of acid matters into the intestines. The symptoms will vary in different individuals, and according to the age of the subject. Colic, tormina, diarrhœa, intussusception, and convulsions are among the consequences which may flow from it. In very many persons a nervous headache is the attendant upon this state of things, which involves, also, a derangement of the biliary secretion. This headache is limited to the forehead, attended by nausea, intolerance of light and sounds, and is apt to terminate very suddenly, with a sensation as if bile were passing the gall-ducts. Our author ascribes the relief experienced under such circumstances, to the flow of the bile into the cœcum, where the soda existing in it corrects the acidity upon which the headache depended. He continues, on this subject:

"It is remarkable that those who suffer least from the derangement of the primary digestive processes, often experience the greatest inconvenience from the derangements of the secondary class, or from their consequences. This is perhaps referable in a certain degree to the fact, that such individuals pay less attention to diet, than those whose condition of stomach obliges them to live more cautiously. As an illustration of this point, we may observe, that we frequently hear such individuals boast that nothing disagrees with their stomach; and the consequence is, that they cannot be persuaded to abstain from the most improper things; but partake of every thing alike that comes in their way. In the prime of life, and in sound constitutions, this state of things goes on for periods varying according to circumstances, and particularly according as individuals are indolent or active. In almost all instances, however, sooner or later, the urine becomes loaded, the liver congested, and more or less of fever and derangement of the stomach and bowels; in short, what is usually called a *bilious* attack, takes place. For this a calomel pill and a black dose are resorted to, and all being apparently righted, the individual resumes his former habits; and after a time again undergoes the same round of changes."

In malarious districts such derangements of the assimilating organs are not only more common, but they are also very often followed or accompanied by ague, rheumatism and neuralgia. The treatment of the primary derangements consists in the use of laxatives, regimen, tonics, and alkalis, and where they are succeeded or complicated, as above, quinine must be superadded, or bleeding, purgatives, or other remedies, demanded by the symptoms.

We should like to follow our author through his elaborate and instructive chapter "on the *pathology of albuminous assimilation*," but having, in the previous article, given a long history, under the name of "Bright's disease," of one of the disorders of the kidney described under this head, we will limit ourselves to a notice of the section on lithic acid.

This substance, "which is derived from the albuminous principles," in a healthy condition of the urine remains transparent after cooling, and even until the spontaneous changes incident to it have commenced. But in certain morbid conditions of the system this acid is rendered incapable of being retained in solution, and is precipitated when the urine becomes cold, sometimes even before, either pure, or in combination with soda or ammonia. So long as it is not deposited in the *warm urine* little danger is to be apprehended, unless a calculus has already formed in the kidney or bladder. When a disposition is shown in the acid to concrete together, or when it is deposited in very large quantity, the formation of a calculus may be feared.

Of the sediments of the urine the yellow is esteemed the least indicative of disease. It is produced in healthy or slightly dyspeptic individuals by errors of diet, changes of weather, and a variety of other circustances disturbing the digestive function. Now and then, perhaps, this deposit is seen in the urine of the healthiest persons. The *red* sediment indicates feverish or inflammatory action, and the deeper the color, generally, the more severe the inflammatory symp-

6 *

toms. The *pink* sediment attends upon certain visceral affections, especially of the liver and spleen. The most perfect specimens Dr. Prout has seen, "have occurred in dropsical cases associated with hepatic disease. When dyspepsia is connected with formidable visceral disease, the urine is occasionally of a bright pink color."

"The *symptoms* local and constitutional accompanying the deposition of lithic acid in the massive condition from the urine, are more or less of pain or uneasiness in the region of the kidneys; with irritation and sense of heat about the neck of the bladder and urethra. There is also a frequent desire to pass the urine, which is voided in small quantities at a time, and without affording the usual relief; the sensation still continuing of something being left behind in the bladder. The digestive functions also, as in most cases where urinary deposites are concerned, are considerably deranged, or very liable to become so; and the patient is very frequently troubled with acidity of the stomach, flatulence, &c., particularly after any little error in diet; as the use of fruits, acescent wines, &c."

The causes of lithic acid sediments may be classed under the three heads of *diet*, *exercise*, and *atmospheric influences*, of which the first is much the most effective, although the want of exercise, especially if conjoined with full or improper diet, must be regarded as a fruitful source. It is an interesting fact, that "many individuals subject to derangements of the general health, seldom feel so well with respect to their health, as when these deposits take place in the urine." This is particularly true in cases of gouty individuals.

Of the treatment, it is not necessary to say much. That must be conducted upon general principles. All the measures which promote the process of assimilation are to be carefully observed and enforced. Our author justly observes, that—

"It cannot be too strongly impressed upon those who suffer from gravelly affections in middle life, that the *cause* of these affections lies deep in the constitution; and that to counteract their distressing effects, perseverance in the appropriate diet, regimen, and medicine, is absolutely necessary.

It is absurd to look for permanent relief in these complaints, by attention to regimen and medicines for a few days or weeks. In obstinate cases, an adherence, more or less strict, according to circumstances, to the principles above stated, should be adopted for months, or even for years, to ensure success."

Our author correctly remarks, in the preface, that the principles of his work include almost every disease to which organized beings are liable, but in our partial notice we shall be obliged to omit many of the subjects. Cystic oxide is one of these, which we are the readier to do because it is a disease of rare occurrence. So, too, the chapter on *the pathology of oleaginous assimilation and secretion*, including the cause of obesity and leanness, and the nature, symptoms, and treatment of gall-stones, will be passed over, while we proceed to give some account of the diseases connected with insoluble incidental matters in the urine. And first of the phosphate of magnesia and ammonia, and the phosphate of lime.

In a healthy condition of the system, these are evolved in a state of solution; but under the influence of disease the balance of the natural affinities is broken, and they are deposited in the urine, and in other parts of the body in the solid form. Dr. Prout thinks the symptoms produced by the triple phosphate of magnesia and ammonia,, and the phosphate of lime, as well as the immediate origin of these two compounds, are different.

"The *triple phosphate of magnesia and ammonia* is usually deposited from the urine in the form of perfectly white shining crystals, and constitutes what is denominated *white gravel;* in contradistinction to the lithic acid crystals, which from their color, is termed *red gravel.* The white form of gravel sometimes occurs alone, but very frequently it alternates with, or is accompanied by, the pale colored lithic amorphous sediments; or the amorphous variety of phosphatic sediment, to be presently described."

Nervous irritability is characteristic of this diathesis, which manifests itself in various ways. In some cases, the symptoms simulate asthma; sometimes spasmodic action of the bowels, borborygmi and flatulence attend; and again the patient has copious, exhausting motions. The symptoms indicative of the deposition of phosphate of lime are not so strongly marked, although partaking of the same irritable character. In the latter a cachetic state of the system is often present, manifested by cutaneous eruptions of a scaly or leprous character. The urinary affection appears to alternate with this eruptive disease, growing better when the disorder of the skin is worse, and *vice versa.*

"The *phosphate of lime* is deposited from the urine in the form of an impalpable amorphous powder, which is generally white; though occasionally it is slightly tinged with the coloring matters of the urine. The properties of the secretion depositing the phosphate of lime are various. In a few instances, I have seen deep colored, acescent urine, of a specific gravity above 1.030, and possessing all the sensible qualities of urine in an exalted degree, deposite phosphate of lime in large quantity; indeed urine depositing this salt is not unfrequently acescent, at least when passed."

These salts, the triple phosphate, and the phosphate of lime, although occasionally, are yet rarely, deposited in separate states. In by far the greater number of cases the earthy deposites consist of a mixture of the two, the properties being varied in every possible degree.

"The phenomena when presented by the urinary deposite consisting of the *mixed phosphates*, vary considerably, according as the one or the other salt abounds, and according, more especially, to the source of the phosphate of lime. When the triple phosphate predominates, the deposite assumes more of the crystalized form; when the phosphate of lime predominates, and is derived from the kidney, the deposite is usually in the form of a white (or yellowish) impalpable powder; or if the phosphate of lime be derived from the coats of the bladder, the deposite usually appears partly in the form of a white powder, and partly in the form of large crystalline granules, enveloped in much tenacous mucus. As in the other form of the deposite, the phenomena presented by the urine abounding in the phosphates is liable to great variations. When the disease is constitutional, and not connected with local causes, as disease of the bladder, &c., the urine is almost always pale colored, and, on the whole voided in greater quantity than natural. The quantity (and of course the quality) is subject to very great and capricious changes, and sometimes, especially by day, the urine is voided in enormous quantity, amounting to actual diuresis. On such occasions the urine is pale colored and of low specific gravity; indeed, I have occasionally seen it perfectly limpid, and scarcely differing in weight from common spring water. At other times, the urine is voided in very small quantity, in which case it is deep colored, and of a specific gravity of 1.025 or higher. When the urine is abundant and of low specific gravity, it is usually free from deposite; on being submitted to heat, however, it generally becomes turbid from a deposition of the phosphates. When voided in small quantity, on the contrary, the urine is often turbid when passed; and in almost all instances, on standing for a time, it deposites the mixed phosphates in abundance. When the bladder is diseased, the urine is generally alkalescent from the beginning; and the earthy deposites are enveloped in large quantities of mucus, which is sometimes tinged with blood. In all instances, however, even if it be slightly acescent at first, it becomes alkalescent on cooling; hence, from its proneness to decomposition, urine depositing the phosphates is apt to become exceedingly offensive in a short time after it is passed."

A deposition of the mixed phosphates is apt to be accompanied by very distressing constitutional symptoms. Great derangement of the chylopoietic viscera is present, attended by universal irritability of the general system. The expression of the countenance becomes sallow and haggard, and, at length, coldness of the legs, anaphrodisia, great languor and depression of the spirits, and other symptoms of extreme debility supervene, such as those which attend upon diabetes.

"An excess of the mixed phosphates in the urine, considered as an idiopathic disease, is not of common occurrence; and the deposition of these salts is much more frequently accompanied by local diseases of the urinary organs. Hence a deposition of the phosphates usually accompanies protracted bladder and prostate affections; and thus superadds too frequently the miseries of stone to the other sufferings of the patient. Even the presence of a stone in the bladder, of whatever it may be originally composed, if permitted to remain, sooner or later produces a deposition of the phosphates; which, by incrusting the stone, adds rapidly to its bulk; and thus increases tenfold the patient's misery."

In the treatment of these affections, diet and regimen are matters of the utmost importance, and our author insists, in regard to the former, that a due proportion of animal food is demanded by the asthenic condition of the vital powers. The diet should consist of solids principally, and he does not object to the use of wine and cider where the individual has been in the habit of drinking them. Whatever disagrees with the stomach is, of course, to be avoided, and, in like manner, as to exercise, whatever fatigues or exhausts, either bodily or mentally, will do mischief. The exhilarating air of the country, agreeable employment, and absence from care, are influences to be invoked in the cure of this complaint, and will often effect it in "those slighter and induced cases, in which the affection is not complicated with local injury." We cannot follow the author into the details of the medical treatment;—he states the general method of it in the following sentence:

"Sedatives, tonics, and antalkaline remedies, therefore, of various kinds, and variously associated, according to the particular symptoms and circumstances of the case constitute the most efficient means we possess of combatting that peculiar condition of the system, accompanied by a deposition of the triple phosphate in the urine; and in general the slighter cases will readily yield to the due administration of these remedies, while the severer and more deep-seated cases will commonly become ameliorated under such a plan of treatment."

Book II is devoted to the consideration of Mechanical Diseases, or *those involving lesions of the kidney and bladder, and particularly from the presence of concretions in these organs.* These subjects, of all that are discussed in the volume before us, are those which have attracted the most general attention, and with the symptoms of which the profession is most familiar. Calculi in the kidneys—the diseases produced by, and liable to be confounded with calculus in these organs—calculi in the bladder—the diseases liable to be confounded or complicated with vesical calculi—hemorrhage from the urinary organs—incontinence and retention of urine—these are affections so clearly marked, and generally so distressing in their nature, that the practitioner is not likely either to disregard them, or to err in his diagnosis. A chapter is devoted to the removal of calculi from the bladder, by solvents, and by the operations of lithotomy and lithotrity. In regard to the first mode, however, the author feels "obliged to confess that in spite of all the boasted light of modern science, the problem remains unsolved, and that we cannot by any known medicinal means, or at least by any known method of directing these means, get rid of a large calculus in the bladder, by other than mechanical expedients." Still, he does not regard the removal of calculi from the bladder without surgical operation, as impossible. On the contrary he thinks that "considerable progress has been made in certain favorable instances, towards the attainment of such object." But he remarks that the introduction of lithotrity has taken away a good deal of the interest which was once felt in this subject, affording as it does, where practicable, a safe mode of removing visceral calculi. Unfortunately, however, the cases are very numerous in which it is not practicable.

The third part of Dr. Prout's work embraces many facts and speculations in physiology which we should be glad to embody in this article, but the few pages left us forbid the attempt. From an inspection of the heads of the several sections it will be perceived how rich a store of information is here provided for the diligent searcher after knowledge of the animal functions. They are as follows: "The ultimate composition and structure of organized bodies, and of their general physical characters, as dependent on their composition;" "alimentary proximate principles;" "the primary processes of assimilation;" "the secondary processes of assimilation;" "the general pathology of the primary and secondary assimilating processes;" "the general composition and properties of the blood;" "the functions of the liver, and composition and relations of the bile to the assimilating processes;" "biliary concretions or gall-stones;" "the functions of the kidneys, and composition and relations of the urine to the assimilating processes;" "urinary calculi, their composition and characteristics;" to which is subjoined "an *appendix* containing tables illustrating the number of fatal cases of diabetes and calculus in different districts in England and Wales; the comparative prevalence and laws of formation, and alteration of different calculous deposites; of the comparative prevalence of calculous affections at different ages and in different sexes; and of the rate of mortality from the operation of lithotomy."

The student who is in quest of the fullest information on any of these topics, afforded by the present state of the science, will find it in this volume. Dr. Prout is not a mere compiler of other men's opinions and discoveries, but an original inquirer, and deep thinker, whose learning in the related sciences imparts uncommon value to his physiological and medical speculations. As a specimen of his manner of solving the difficult problems of vital action, we give the following extract, in which an attempt is made to account for certain changes occurring during the process of assimilation:

"Supposing the chlorine or muriatic acid found in the stomach to be derived from the common salt existing in the blood and in the stomach, another question arises,—what becomes of the soda from which the muriatic acid has been separated? The soda remains behind, or is absorbed into the mass of blood, and a portion of it no doubt is requisite to preserve the weak alkaline condition essential to the fluidity of the blood. But the larger part of this soda is probably directed to the liver, and is elicited with the bile in the duodenum; where it is thus again brought into union with the acid, which had been separated from the blood in the stomach. These observations, illustrating the importance of common salt in the animal economy, seem to explain, in a satisfactory manner, that instinctive craving after this substance, which is shown by animals.

"Admitting that the decomposition of the salt of the blood,

&c., is owing to the immediate agency of a modification of electricity, we have in the principal digestive organs a kind of galvanic apparatus, of which the mucous membrane of the stomach and intestinal canal, generally, may be considered as the acid or positive pole, while the hepatic system may, on the same view, be considered as the alkaline or negative pole."*

So, in the following quotation, we have the function of the liver and kidneys explained, with the phenomena which result from their perverted action:

"The liver and the kidneys (as well as certain minor glandular appartus) either in virtue of the polar arrangements above mentioned, or of some other (vital) property, must, in a state of health, possess the *function of secreting* from the blood those peculiar principles adapted for their respective operations; and of producing such further changes in them as the animal economy may require. The changes produced by the liver on the principles to be eliminated by that gland, are in some degree of an organizing kind; that is, the principles separated retain some of their vitality for ulterior purposes; while the changes produced by the kidneys on the principles designed to be removed from the system by these glands, are, in a state of health, without exception, of a disorganizing kind—that is, every thing passed from the kidneys is denuded of its vitality, which is carefully retained as it were in the system. The liver, therefore, may be said to possess an *organizing*, the kidneys a *disorganizing function.* This deduction is illustrated by what takes place in the diseases of the liver and kidneys. Thus, when the liver is diseased, its selecting or organizing functions are impaired or lost; and instead of selecting, and further changing into bile those principles, which the welfare of the economy requires should be removed from the blood and employed elsewhere, such principles are retained in the system; or if they do pass through the liver and are separated by that organ, they are imperfect-

---

* "This notion or opinion, which was first advanced by me many years ago, seems to have lately received some confirmation from the experiments of Matteuci, who found that when the liver and stomach of a rabbit were connected with the platinum ends of the wires of a delicate galvanometer, a deviation of the needle took place amountlng to fifteen or twenty degrees. This action became very feeble, or entirely ceased, after the death of the animal; hence he inferred that it depended on the vital action of the organs, and not on the difference of the chemical properties of their secretions."

ly adapted for their ulterior functions; and thus in both ways great derangements of the health are the consequence.—Again, when the kidneys are diseased, their selecting and disorganizing functions are impaired or lost; and the deleterious principles of the blood, (e. g. the urea) are no longer selected in preference for separation from that fluid; while the superflous or effete albuminous principles, which in the healthy kidney would have been selected and converted into the lithate of ammonia, either remain in the blood, or pass through the kidneys unchanged."

Here we must close our notice of this work. Many subjects have been passed over altogether, while others have been noticed so briefly, that only a very partial knowledge of them can be acquired from this article. Enough, however, has been said to convey to our readers an idea of the scope and character of this treatise on "*Stomach and Renal diseases.*" "Some books," says Lord Bacon, "are to be read—others are to be read and *digested.*" It is plain, that the work before us belongs to the latter class. It is a work to be studied, and one which will amply repay the diligent student for all the time bestowed upon it. The physician in extensive practice, too, will find it most valuable as a book of reference, in many of the obscure cases of disease which he must often encounter. We are glad that a new edition of it has been published in this country, and cannot doubt that it will be eagerly sought after by the profession. No diseases, we believe, have been more neglected by the physicians of our country than many of that important class to the study of which Dr. Prout has devoted a long life; certain, we are, that they have no where been treated with greater clearness and ability than in the volume which embodies his large and matured experience. Y.

